# Integrative analysis of the roles and prognostic value of RNA-binding proteins in papillary renal cell carcinoma

**DOI:** 10.1007/s12672-026-05437-8

**Published:** 2026-06-19

**Authors:** Tian Niu, Qicong Li, Ao Zhang

**Affiliations:** Department of Urology, The Affiliated Hospital of Changchun University of Chinese Medicine, Changchun, China

**Keywords:** Papillary renal cell carcinoma, RNA-binding proteins, Bioinformatics, Prognostic model, Survival

## Abstract

RNA-binding proteins (RBPs) serve essential roles in various cancer types, but their functions in papillary renal cell carcinoma (pRCC) have not been elucidated to date. In our work, differentially expressed RBPs in pRCC were identified after acquisition of RNA-sequencing and clinical data related to pRCC from The Cancer Genome Atlas database(TCGA). Functional enrichment analysis and protein interaction network analysis, along with univariate and multivariate Cox regression analyses, were performed to uncover potential biological effects of the identified RBPs and screen the hub RBPs for pRCC prognosis. We identified 251 up-regulated and 129 down-regulated RBPs, and filtered out seven hub RBPs, namely, SRSF8, CD3EAP, HBS1L, ELAC2, MRPL34, NOP2 and IGF2BP2, for their prognostic relevance. A prognostic risk score model for overall survival of pRCC patients was constructed based on the seven hub RBPs. Further analysis showed that the low-risk group had higher survival rate than the high-risk group in both training and validation cohorts. The predictive accuracy was verified in the Human Protein Atlas database.In addition, we introduced the GSE15641 dataset from the Gene Expression Omnibus (GEO) database for independent external validation, and confirmed the expression levels of HBS1L, MRPL34 and IGF2BP2 through real-time quantitative PCR (RT-qPCR) and Western blotting (WB) using human renal tubular epithelial cell line HK-2 and human papillary renal cell carcinoma cell line Caki-2. In pRCC, CD3EAP was significantly elevated, while ELAC2, IGF2BP2, MRPL34, SRSF8 and HBS1L were significantly reduced. There was no significant difference between tumor and normal tissues in NOP2 expression. Risk score and tumor grade were independent prognostic factors associated with overall survival. In addition, we established a nomogram based on the seven prognostic RBPs to help predict overall survival at 1–3 years. In conclusion, seven differentially expressed hub RBPs were identified as potential prognostic biomarkers for pRCC. Our prognostic model might serve as a support for better treatment decision-making. Our work could provide potential new ideas for diagnosis and research on targeted drugs for pRCC.

**Supplementary Information:**

The online version contains supplementary material available at https://doi.org/10.1007/s12672-026-05437-8.

## Introduction

Renal cell carcinoma (RCC) is by far the most lethal kind of urinary tract cancer and constitutes almost 3% of adult malignancies, with annually increasing incidence and mortality [44, 49, 74]. Papillary renal cell carcinoma (pRCC) is the second most frequent subtype of RCC following clear cell RCC and represents approximately 15–20% of identified RCC cases [40]. Although clear cell type is much more common, papillary carcinoma tissue or architecture is present in nearly 40% of multifocal RCC cases [10, 38]. pRCC patients are more likely to receive an accurate diagnosis than clear cell cases [13]. pRCC most often metastasizes to the lung, liver, brain and bone, just as clear cell RCC does, but with an increased involvement of lymph nodes according recent findings [6, 36]. Papillary type is considered clinically less aggressive than clear cell type, but its advanced or metastatic cases would face significantly higher mortality [31]. Radical surgery is still now the only curative treatment option for pRCC. Therefore, further exploration of the molecular mechanisms of pRCC is needed to help obtain more satisfactory screening, diagnosis, treatment and prognosis for pRCC patients.

RNA-binding proteins (RBPs) are multi-faceted proteins that constitute one or multiple RNA-binding domains capable of binding RNA. To date, 1542 RBPs accounting for slightly over 7% of all protein-coding genes, have been identified by whole-genome sequencing and bioinformatics analysis [21]. These RBPs serve essential post-transcriptional roles in regulating RNA splicing, mRNA stabilization, cytoplasmic mRNA export, localization and translation [5, 17]. Neurodegenerative and cardiovascular diseases, as demonstrated, are associated with RBP abnormalities [3, 41]. Most intriguingly, RBPs have been found or largely demonstrated to be dysregulated or involved in various human malignancies [61]. Overall, it is tempting to postulate that the way RBPs regulate gene expression may help uncover the mechanism of tumorigenesis.

In recent years, mounting in-depth studies have indicated the involvement of RBPs in tumorigenesis. G3BP1 is present in various types of tumors and could promote cell proliferation and metastasis and inhibit cell apoptosis via regulation of the Ras, Src/FAK, TGF- beta/Smad and p53 signaling pathways [67]. In non-small-cell lung cancer, SART3 overexpression could affect cell cycle progression via its upregulation on miR-34a expression and downregulation on miR-34a target genes CDK4/6 [48]. HuR could regulate the expression of several urinary tumor-associated molecules by post-transcriptional control of mRNA trafficking, decay and translation [68]. Breast cancer cell proliferation would be attributable to post-transcriptional control of proliferation-related genes E2F8 and SKP2 by NONO [30], and to RPL11/MDM2-mediated p53 activation by RRS1 [51]. In addition, RRS1 could accelerate proliferation and invasion of retinoblastoma cell through its activating the AKT/mTOR signaling [64], and its downregulated expression by miR-148a may inhibit the proliferation, migration and invasion of and facilitate the apoptosis of cervical cancer cells [71]. These findings strongly suggest that RBPs would work as reliable molecular biomarkers and therapeutic targets for cancers. Thus, in this study, we performed an integrative bioinformatics analysis of the potential prognostic value or biological functions of RBPs in pRCC to better understand the development and progression of pRCC, and further established a prognostic risk score model for its overall survival.

## Materials and methods

**Fig. 1:**
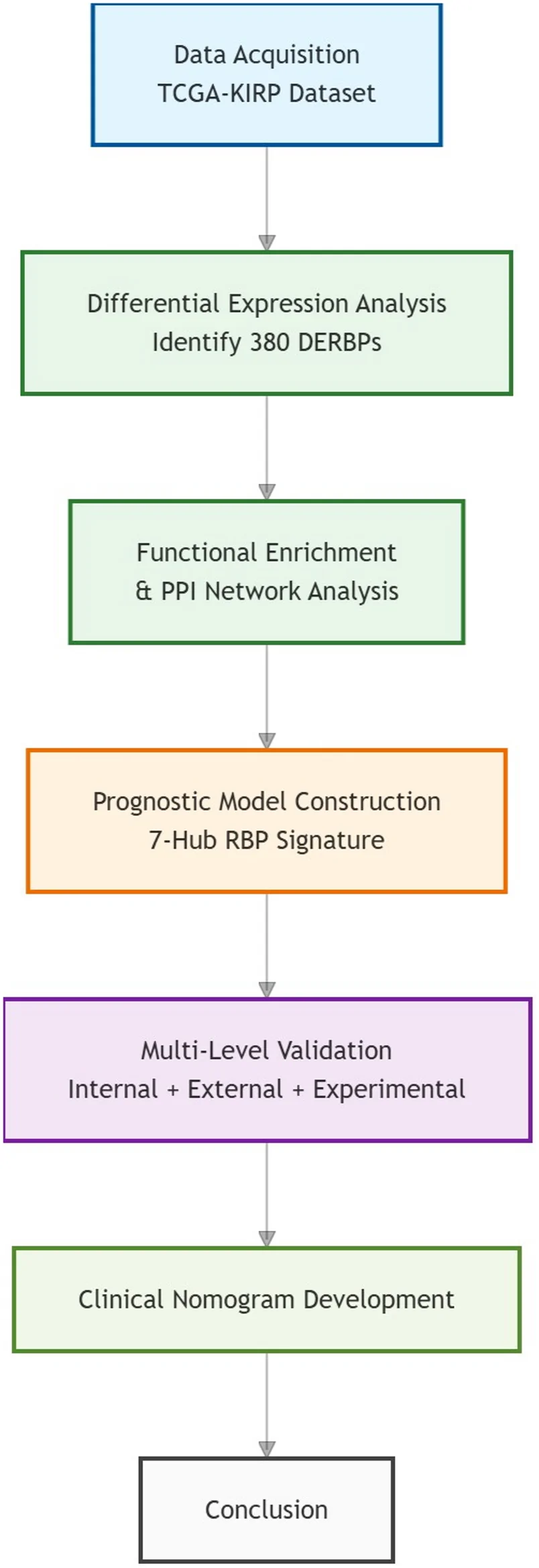
Study flow chart

The overall workflow of this study is illustrated in Fig. 1.

### Data acquisition and identification of differentially expressed RBPs

The RNA-seq transcription data and relevant clinical information of 289 pRCC samples and 32 normal renal tissue samples were downloaded from the Cancer Genome Atlas database (TCGA) (portal: https://portal.gdc.cancer.gov/). The included data were arranged using a homemade Perl script to obtain an array with Ensembl IDs as the row names and sample names as the column names. The row names of the array were then converted to gene names. Raw gene array data from TCGA was normalized using the LIMMA package from the R Bioconductor software (downloaded from the portal: http://www.bioconductor.org/packages/release/bioc/html/limma.html) to obtain relevant RBPs for screening the gene expression profile. Differentially expressed RBPs (DERBPs) were identified with the LIMMA package and the cut-off criteria of |Log_2_ fold-change| > 0.5 and false discovery rate (FDR) < 0.05. The pheatmap and ggplot2 packages in R language were utilized to generate heatmap and volcano plot of DERBPs, respectively.

### Functional enrichment analysis of DERBPs

Gene Ontology (GO) term and Kyoto Encyclopedia of Genes and Genomes (KEGG) pathway enrichment analyses were conducted to further reveal the biological functions of the DERBPs using R packages. GO contained three domains, molecular function, cellular component and biological process. A GO term with *P* value < 0.05 and FDR < 0.05 or a KEGG pathway with *P* value < 0.05 was considered a statistically significant enrichment.

### Construction of protein-protein interaction network

The selected DERBPs were uploaded to the online STRING database (portal: http://www.string-db.org/) to construct the protein-protein interaction (PPI) network. Cytoscape software (version 3.8.2) (portal: http://www.cytoscape.org/) was applied to visualize the PPI network. Gene cluster modules were identified using the MCODE plugin in Cytoscape. Node score cutoff > 4, *P* value < 0.05 and FDR < 0.05 were set as the threshold.

### Construction and evaluation of prognostic model for pRCC

The DERBPs associated with pRCC prognosis were screened via univariate and multivariate Cox regression analyses with P value < 0.05 as the threshold for significance. On this base, a prognostic risk score model was constructed with the following calculation formula, risk score = *coef*1 * *Exp*1 + *coef*2 * *Exp*2 + *coef*x. In the formula, Exp refers to the expression value of a RBP gene, and coef represents the coefficient of corresponding Cox regression. Furthermore, based on the hazard ratio (HR) values, the patients were divided into low-risk (HR < 1) and high-risk (HR > 1) groups, and the Kaplan Meier estimator was applied to estimate the probability of survival of the high-risk and low-risk groups over time. The time-dependent ROC (receiver operating characteristic) curve analysis was performed to evaluate the diagnostic performance to further assess the prognostic power of these biomarkers. Univariate and multivariate analyses of relevant clinical characteristics (e.g., patient age and sex, and tumor grade) were also performed to assess independent prognostic factors associated with overall survival in pRCC patients. Besides, a nomogram containing all prognosis-related RBP genes was constructed to quantitatively predict the survival time of pRCC patients. The expression of each RBP gene in the nomogram corresponded to a score (0–100). The total risk score was calculated as the sum of all the scores for the RBP genes and applied to predict 1/2/3-year survival probability for pRCC patients.

### Validation of RBP-related gene signature

We further verify the expression of the hub prognosis-related RBP genes using immunohistochemistry data from the Human Protein Atlas online database (portal: http://www.proteinatlas.org/).

### External validation using GEO dataset

Transcriptomic data of the GSE15641 dataset, including 32 normal renal tissues and 32 pRCC tissues, were downloaded from the Gene Expression Omnibus (GEO) database (https://www.ncbi.nlm.nih.gov/geo/). The data were normalized using the limma package in R language. The expression levels of the seven hub prognostic RBPs were extracted, and a volcano plot and expression heatmap were generated. Receiver operating characteristic (ROC) curve analysis was performed to evaluate the diagnostic efficacy of each hub RBP for pRCC, and the area under the curve (AUC) values were calculated.

### Cell lines and experimental validation

#### Cell culture

The human renal tubular epithelial cell line HK-2 and human papillary renal cell carcinoma cell line Caki-2 were purchased from the Cell Bank of the Chinese Academy of Sciences (Shanghai, China). Cells were cultured in DMEM medium supplemented with 10% fetal bovine serum (FBS), 100 U/mL penicillin and 100 µg/mL streptomycin, and incubated at 37 °C in a humidified atmosphere with 5% CO₂.

#### Real-time quantitative PCR (RT-qPCR)

Total RNA was extracted from cells using Trizol reagent, and reverse transcribed into cDNA using a reverse transcription kit. GAPDH was used as the internal reference gene, and qPCR reactions were performed using the SYBR Green fluorescent quantitative kit. The primer sequences were as follows: HBS1L forward 5’-AGTGGCTGATGAAGGTGCTG-3’, reverse 5’-TCCAGCTTGTAGGTGATGGC-3’; MRPL34 forward 5’-GCTGCTGAAGATGGTGGTGA-3’, reverse 5’-CCAGGTGATGGTGATGGTGT-3’; IGF2BP2 forward 5’-ACGAGTGGCTGATGAAGGTG-3’, reverse 5’-TGGCTGGTGATGGTGATGGT-3’; GAPDH forward 5’-GAGTCAACGGATTTGGTCGT-3’, reverse 5’-TTGATTTTGGAGGGATCTCG-3’. The reaction conditions were: pre-denaturation at 95 °C for 30 s; 40 cycles of denaturation at 95 °C for 5 s and annealing at 60 °C for 30 s. The relative gene expression levels were calculated using the 2^-ΔΔCt method.

#### Western Blotting (WB) assay

Total protein was extracted from cells, and the protein concentration was determined using the BCA method. Equal amounts of protein were separated by SDS-PAGE electrophoresis and transferred to PVDF membranes. After blocking with 5% skim milk at room temperature for 1 h, the membranes were incubated with primary antibodies (HBS1L, MRPL34, IGF2BP2 and GAPDH, all diluted at 1:1000) overnight at 4 °C. The membranes were washed three times with TBST for 10 min each, and then incubated with HRP-conjugated secondary antibodies (diluted at 1:5000) at room temperature for 1 h. After washing three times with TBST, the membranes were developed using an ECL chemiluminescence kit. The gray values of the bands were quantified using ImageJ software.

### Statistical analysis

R software (version 4.0.0) was used for statistical analysis. The LIMMA package was applied to obtain relevant RBPs within the included data from TCGA database and identify DERBPs. Univariate and multivariate Cox regression analyses were used to screen the DERBPs associated with pRCC prognosis. The Kaplan Meier method was applied to analyze the overall survival of the high-risk and low-risk groups. The time-dependent ROC curve analysis was performed to evaluate the diagnostic performance. The independent prognostic factors associated with overall survival were assessed via univariate and multivariate analyses of relevant clinical characteristics of pRCC patients. *P* values < 0.05 indicated statistical significance.

## Results

### Three hundred and eighty DERBPs in pRCC

**Fig. 2:**
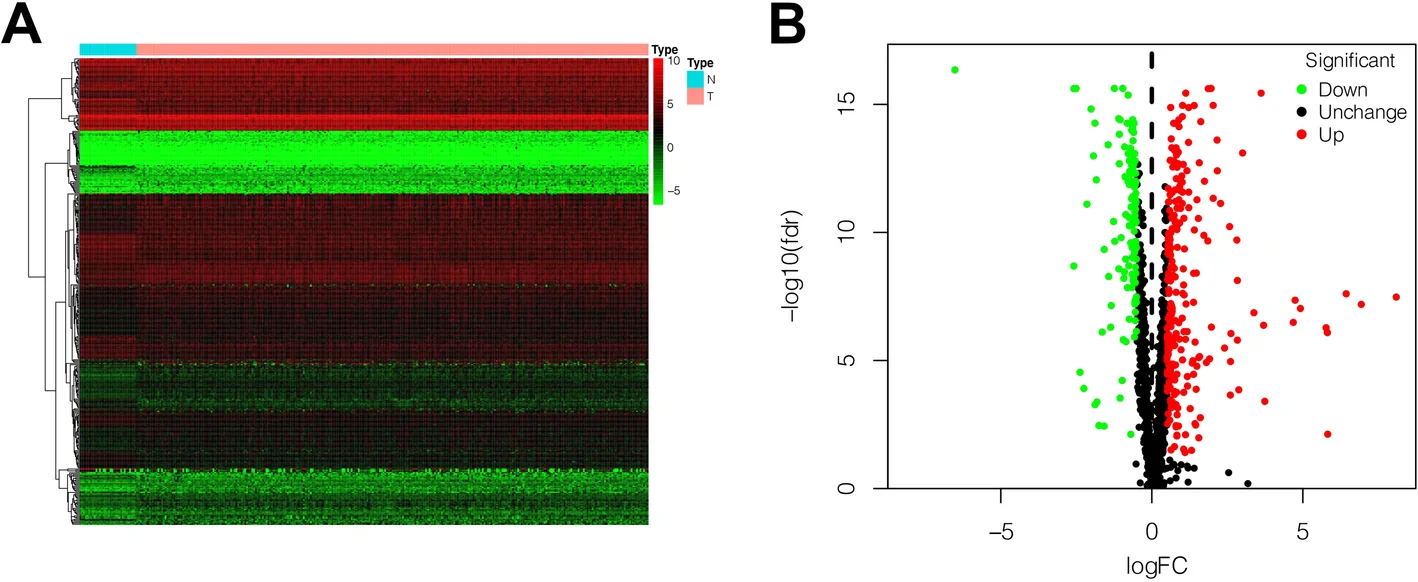
Identification of differentially expressed RNA-binding proteins (DERBPs) in papillary renal cell carcinoma (pRCC).(A) Heatmap showing the expression profiles of 380 DERBPs in 289 pRCC tissues and 32 normal renal tissues from the TCGA database. Red indicates upregulated expression, and blue indicates downregulated expression.(B) Volcano plot visualizing the DERBPs. The cut-off criteria were |log2 fold change| > 0.5 and false discovery rate (FDR) < 0.05. Red dots represent significantly upregulated RBPs, green dots represent significantly downregulated RBPs, and black dots represent non-differentially expressed genes

A total of 1385 relevant RBPs were obtained after analysis of 289 pRCC tissue samples and 32 normal renal tissue samples downloaded from the TCGA database. Further analysis identified 380 significant DERBPs. Of the 380 RBPs, 251 were up-regulated and 129 ones were down-regulated in expression. The heatmap and volcano plot for the identified DERBPs in pRCC were plotted as shown in Fig. 2A and B.

### Functionally enriched GO term and KEGG pathways in pRCC

Functionally enriched GO term and KEGG pathways of the DERBPs were obtained using R packages. The up- and down-regulated RBPs were analyzed separately and the results are shown in Table 1.

**Table 1:** GO term and KEGG pathway enrichment analyses of differentially expressed RBPs

	GO term	*P* value
**Down-regulated RBPs**		
Biological process	Regulation of translation	2.71E-20
	Regulation of cellular amide metabolic process	1.46E-18
	RNA splicing	8.39E-18
	RNA splicing, via transesterification reactions with bulged adenosine as nucleophile mRNA splicing, via spliceosome	5.84E-17
	RNA splicing, via transesterification reactions	6.97E-17
	RNA phosphodiester bond hydrolysis	9.44E-15
	RNA catabolic process	3.94E-13
	Nucleic acid phosphodiester bond hydrolysis	1.75E-12
	Ribonucleoprotein complex subunit organization	2.91E-12
Cellular component	Cytoplasmic ribonucleoprotein granule	2.17E-18
	Ribonucleoprotein granule	5.16E-18
	P granule	1.43E-07
	Germ plasm	1.43E-07
	Pole plasm	1.86E-07
	Spliceosomal complex	4.26E-07
	Chromatoid body	1.10E-06
Molecular function	Catalytic activity, acting on RNA	8.46E-21
	Translation regulator activity	1.39E-19
	Translation regulator activity, nucleic acid binding	3.60E-17
	Translation factor activity, RNA binding	6.62E-16
	Ribonuclease activity	5.47E-14
	Ribonucleoprotein complex binding	5.32E-13
	Nuclease activity	1.85E-10
	Single-stranded RNA binding	5.48E-10
	Translation initiation factor activity	2.24E-09
	Double-stranded RNA binding	2.85E-09
KEGG pathway	mRNA surveillance pathway	1.30E-06
	Nucleocytoplasmic transport	2.67E-06
	Protein export	0.007722361
	Spliceosome	0.010162591
	RNA degradation	0.010654811
**Up-regulated RBPs**		
Biological processes	ncRNA metabolic process	1.37E-40
	ncRNA processing	1.85E-38
	Ribonucleoprotein complex biogenesis	4.23E-38
	RNA splicing	2.30E-34
	RNA catabolic process	8.77E-30
	RNA phosphodiester bond hydrolysis	1.76E-28
	Ribosome biogenesis	2.91E-27
	mRNA catabolic process	4.92E-23
	RNA modification	2.49E-21
	Nucleic acid phosphodiester bond hydrolysis	8.11E-21
Cellular component	Ribosome	2.83E-17
	Ribosomal subunit	9.50E-17
	Spliceosomal complex	1.53E-16
	Large ribosomal subunit	3.09E-15
	Cytosolic ribosome	1.44E-14
	Cytosolic large ribosomal subunit	3.56E-12
	Spliceosomal snRNP complex	7.84E-11
Molecular function	Catalytic activity, acting on RNA	1.37E-46
	Ribonuclease activity	3.89E-22
	Nuclease activity	5.03E-18
	Catalytic activity, acting on a tRNA	2.63E-16
	Structural constituent of ribosome	3.45E-16
	Endonuclease activity	6.61E-14
	Endoribonuclease activity	2.32E-13
	Translation regulator activity	5.61E-11
	mRNA 3’-UTR binding	8.40E-11
KEGG pathway	Ribosome	4.20E-16
	Coronavirus disease - COVID-19	1.02E-13
	Spliceosome	4.15E-11
	RNA degradation	5.45E-08
	mRNA surveillance pathway	4.33E-07
	Nucleocytoplasmic transport	1.17E-06
	Ribosome biogenesis in eukaryotes	1.27E-06
	RNA polymerase	0.000700749
	Legionellosis	0.00602467520395911

RBPs, RNA-binding proteins; GO, gene ontology; KEGG, Kyoto Encyclopedia of Genes and Genomes

In biological process, the up-regulated RBPs were significantly enriched in processing and metabolic process of ncRNA, splicing, catabolic process and modification of RNA and its phosphodiester bond hydrolysis, ribosome biogenesis, ribonucleoprotein complex biogenesis, mRNA catabolic process, and nucleic acid phosphodiester bond hydrolysis. Significant enrichment of down-regulated RBPs was seen in the regulation of translation and cellular amide metabolic process, mRNA splicing, splicing and catabolic process of RNA and its phosphodiester bond hydrolysis, nucleic acid phosphodiester bond hydrolysis, and ribonucleoprotein complex subunit organization.In terms of cellular component, significant enrichment of up-regulated RBPs was found in ribosome and its subunit, large ribosomal subunit, cytosolic ribosome, cytosolic large ribosomal subunit, spliceosome and its snRNP complex. The down-regulated RBPs were significantly enriched in cytoplasmic ribonucleoprotein granule, ribonucleoprotein granule, P granule, germ plasm, pole plasm, spliceosome and chromatoid body.As to molecular function, the up-regulated RBPs were significantly enriched in catalytic activity for RNA and tRNA, the activity of ribonuclease, nuclease, endonuclease and endoribonuclease, structural constituent of ribosome, translation regulator activity, mRNA 3’-UTR binding. Significant enrichment of down-regulated RBPs was found in catalytic activity for RNA, the activity of ribonuclease, nuclease and translation initiation factor, translation regulator activity for nucleic acid binding, translation factor activity for RNA binding, ribonucleoprotein complex binding, and single-stranded and double-stranded RNA binding.

In addition, KEGG pathway analysis showed that the up-regulated RBPs were mainly enriched in ribosome, coronavirus disease, spliceosome, RNA degradation, mRNA surveillance pathway, nucleocytoplasmic transport, ribosome biogenesis in eukaryotes, RNA polymerase, and legionellosis. The down-regulated RBPs were mainly enriched in mRNA surveillance pathway, nucleocytoplasmic transport, protein export, RNA degradation, and spliceosome.

### Key modules of PPI network and enriched GO term in biological process

**Fig. 3:**
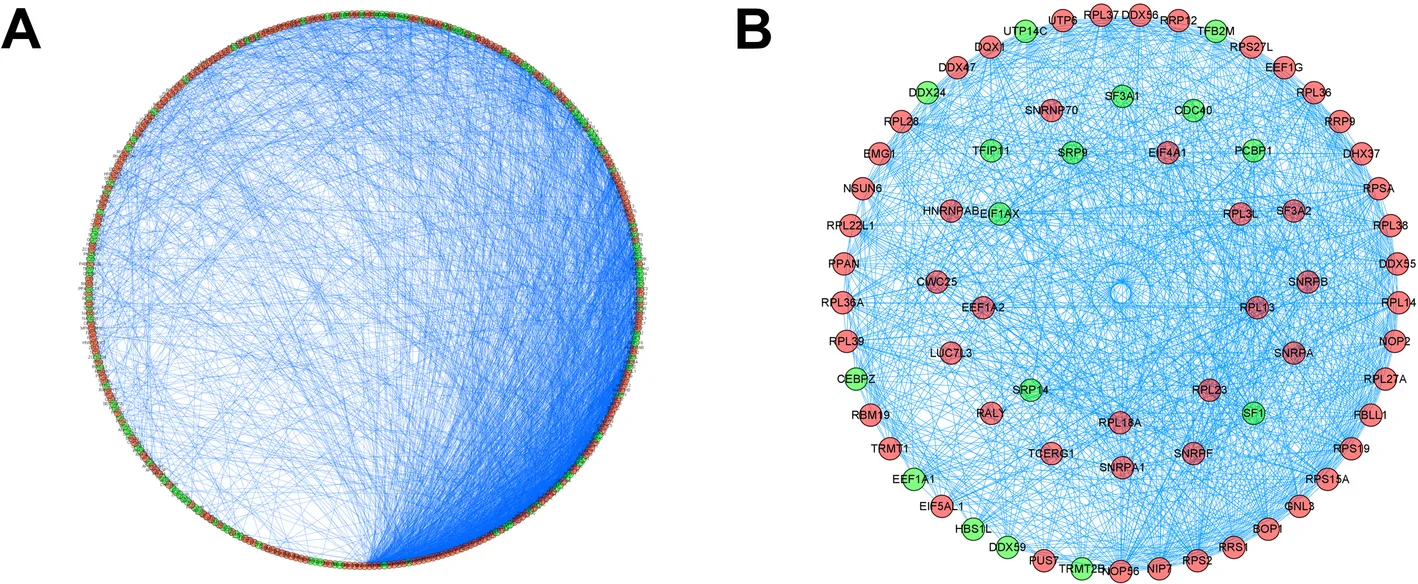
Protein-protein interaction (PPI) network analysis of DERBPs.(A) PPI network of the 380 DERBPs constructed using the STRING database (v11.5). The network contains 351 nodes and 2996 edges. Red nodes represent upregulated RBPs, and green nodes represent downregulated RBPs. Node size corresponds to the degree of interaction.(B) Key gene cluster modules identified from the PPI network using the MCODE plugin in Cytoscape (node score cutoff > 4, *P* < 0.05, FDR < 0.05). Three highly interconnected modules were identified, containing 70 key RBPs in total

The well-constructed PPI network contained 351 gene nodes and 2996 edges (Fig. 3A). Key gene cluster modules were further identified from the PPI network (Fig. 3B). The GO term enrichment analysis for the RBPs in the key modules showed that the RBPs were involved in RNA splicing, RNA catabolism, protein export, rRNA processing, mRNA catabolism, nuclear RNA export, ncRNA metabolism, rRNA metabolism, rRNA binding, tRNA binding and snRNA binding.

**Fig. 4:**
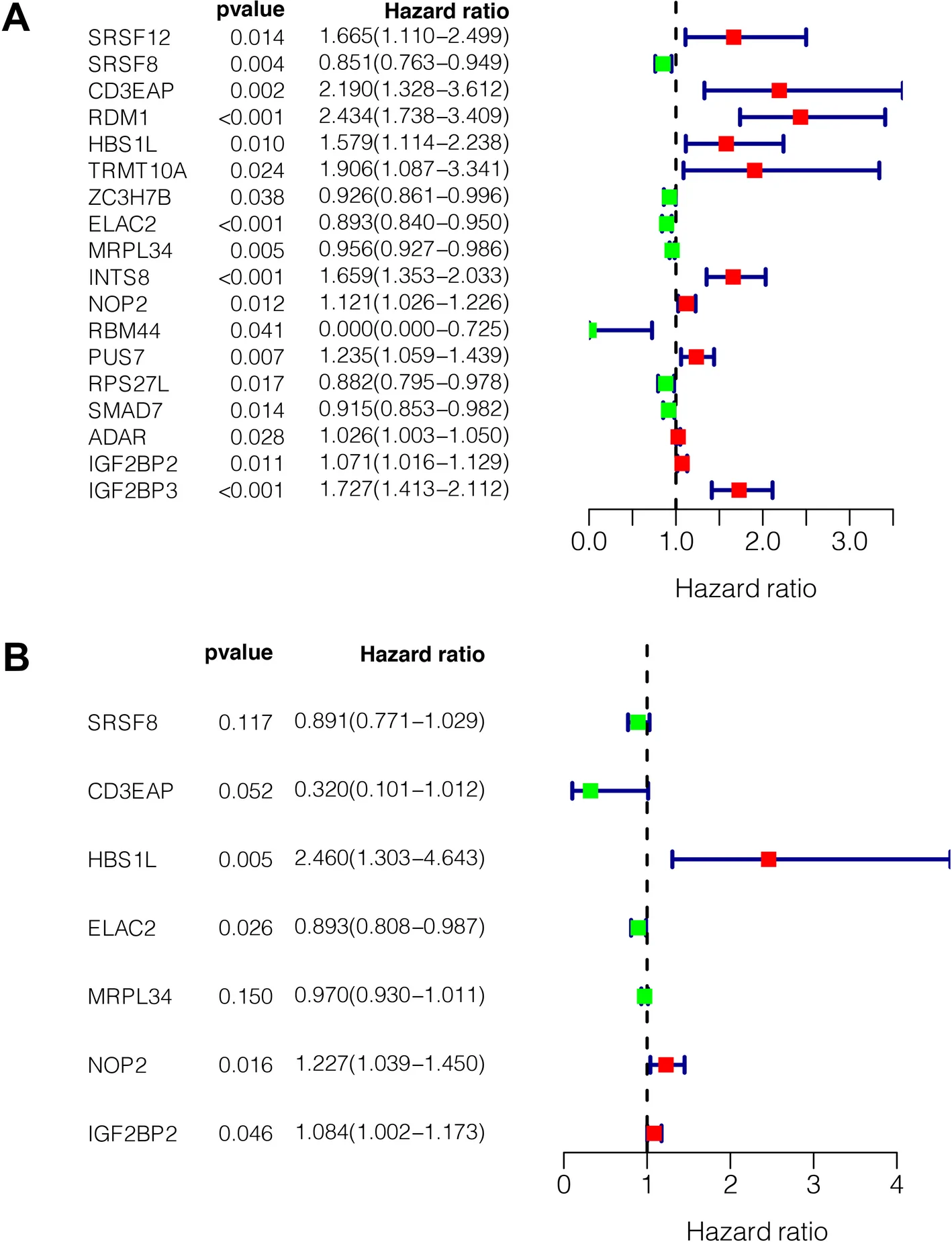
Identification of prognostic hub RBPs using Cox regression analysis. (A) Univariate Cox regression analysis forest plot showing the association between 18 candidate RBPs and overall survival (OS) in the TCGA training cohort. Hazard ratios (HR) and 95% confidence intervals (CI) are shown. *P < 0.05 was considered statistically significant. (B) Multivariate Cox regression analysis forest plot identifying seven independent prognostic hub RBPs. The coefficients from this analysis were used to construct the prognostic risk score model

### Seven pRCC prognosis-associated hub RBPs

Seventy significant DERBPs were screened from the PPI network. With the univariate Cox regression analysis, 18 RBPs associated with pRCC prognosis were obtained out of the 70 RBPs (Fig. 4A). Further with multivariate Cox regression analysis, seven hub RBPs (SRSF8, CD3EAP, HBS1L, ELAC2, MRPL34, NOP2 and IGF2BP2) were finally obtained to assess their association with survival time in pRCC patients (Fig. 4B). These seven hub RBPs could be utilized as independent predictors of pRCC.

### Prognostic risk score model and survival analysis

**Fig. 5:**
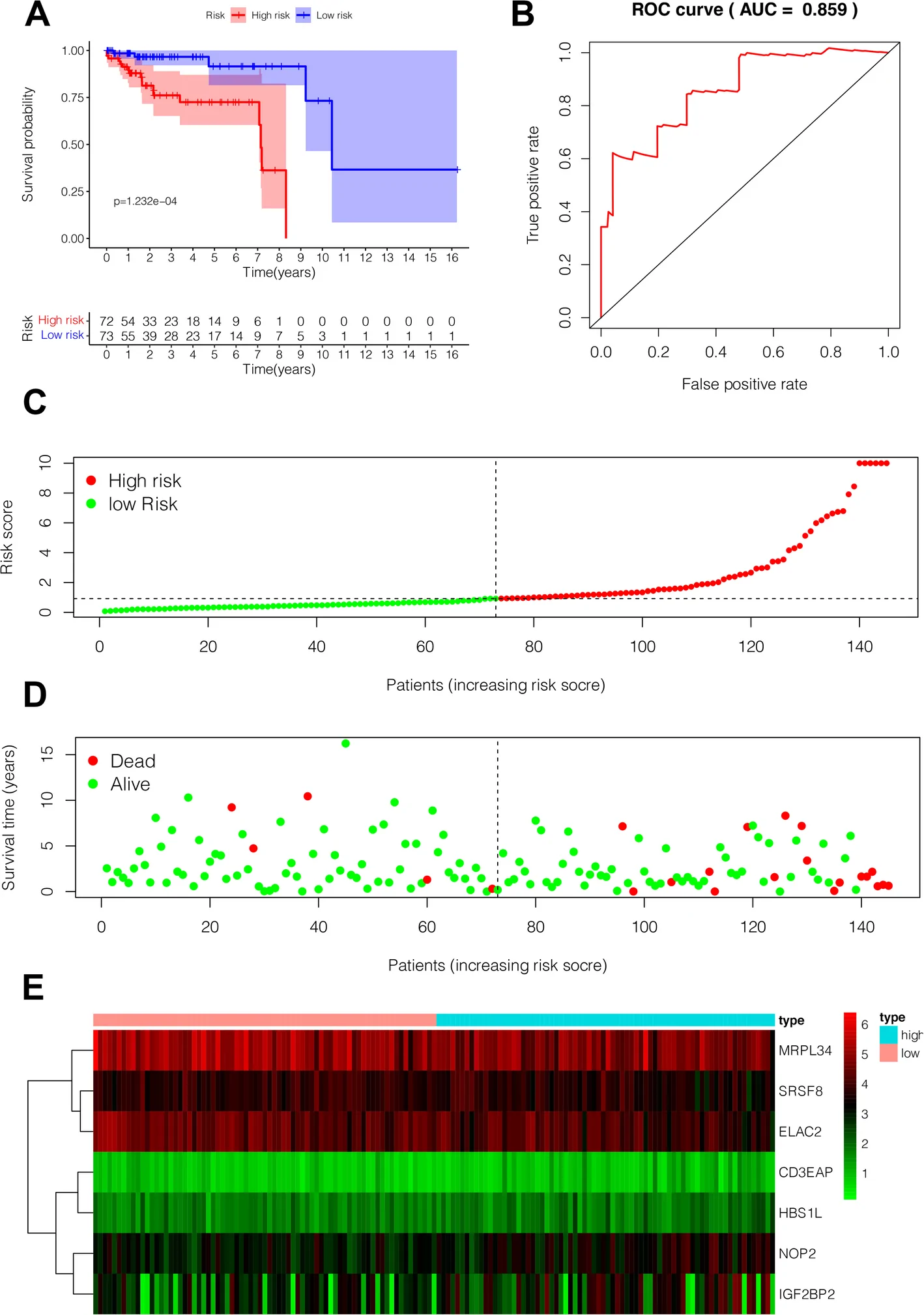
Prognostic performance of the 7-RBP risk score model in the TCGA training cohort (*n* = 145). (A) Kaplan-Meier survival curve comparing OS between patients in the high-risk group (*n* = 72) and low-risk group (*n* = 73), divided by the median risk score. The log-rank test was used to compare survival differences. (B) Time-dependent receiver operating characteristic (ROC) curve evaluating the predictive accuracy of the risk score model for 3-year OS. The area under the curve (AUC) value is indicated. (C) Distribution of risk scores across patients in the training cohort. (D) Scatter plot showing the survival status of patients corresponding to each risk score. Red dots represent deceased patients, and blue dots represent alive patients. (E) Heatmap showing the expression patterns of the seven hub RBPs in the high-risk and low-risk groups

**Fig. 6:**
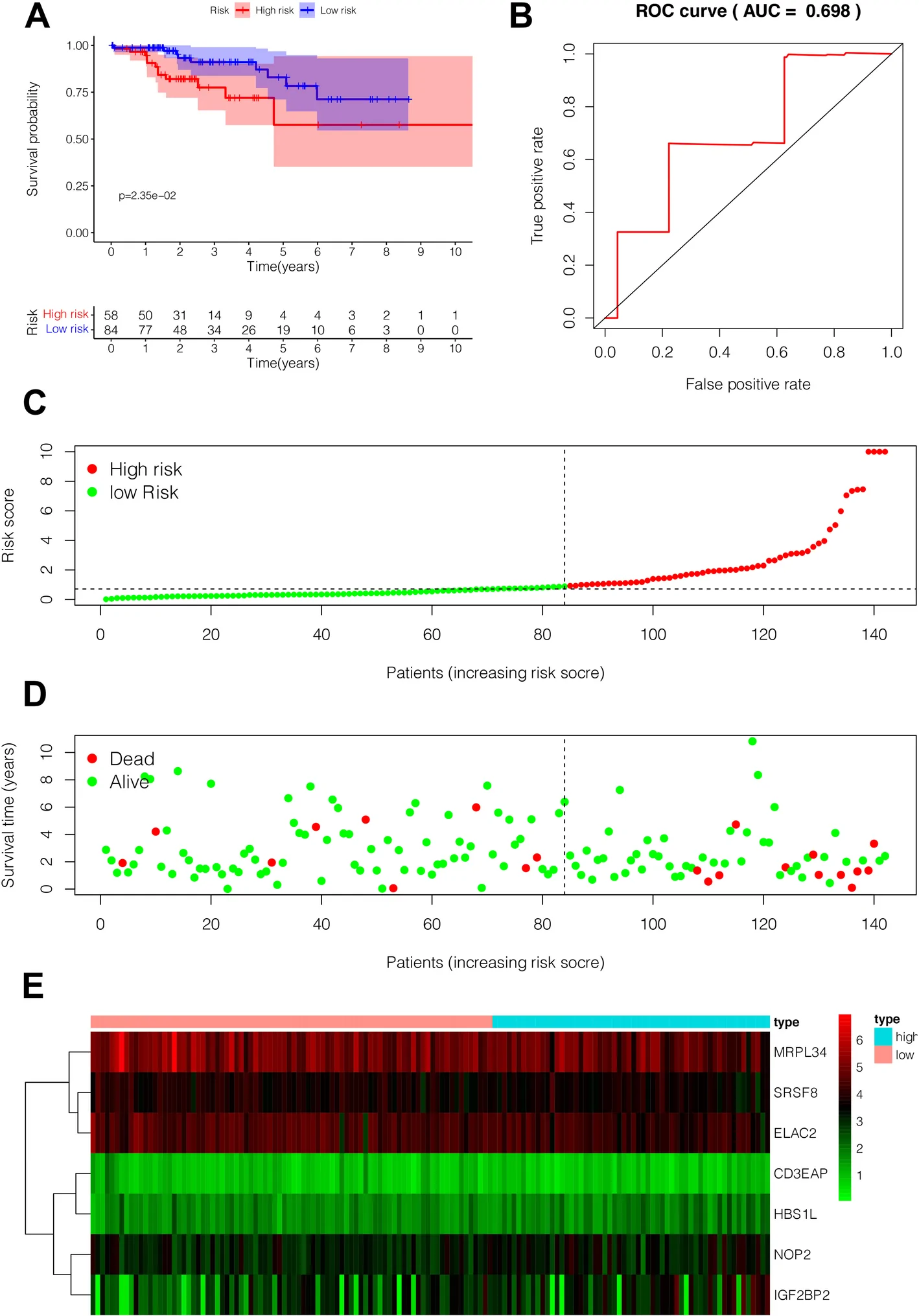
Validation of the 7-RBP risk score model in the TCGA internal validation cohort (*n* = 142). (A) Kaplan-Meier survival curve comparing OS between the high-risk group (*n* = 71) and low-risk group (*n* = 71) using the same median risk score cutoff as the training cohort. (B) Time-dependent ROC curve evaluating the predictive accuracy of the model for 3-year OS in the validation cohort. (C) Distribution of risk scores across patients in the validation cohort. (D) Scatter plot showing the survival status of patients corresponding to each risk score.(E) Heatmap showing the expression patterns of the seven hub RBPs in the validation cohort

The seven hub RBPs were used to construct a prognostic risk score model was constructed, and each patient would receive a risk score for later assessment of the model’s predictive power. The risk scoring formula of the model is as followed: risk score = (0.1158 * *Exp* SRSF8) + (1.1394 * *Exp* CD3EAP) + (0.1131 * *Exp* ELAC2) + (0.0309 * *Exp* MRPL34) + (−0.9002 * *Exp* HBS1L) + (−0.2046 * *Exp* NOP2) + (−0.0803 * *Exp* IGF2BP2). To evaluate the predictive power of the model, 287 patients from the TCGA database were randomly divided into a training cohort (*n* = 145) and a validation cohort (*n* = 142). The patients in the training cohort were divided into high- and low-risk groups according to the median risk score. Survival analysis was conducted and survival curve for the high- and low-risk groups was plotted using R packages (Fig. 5A). We found the survival rate in the low-risk group was higher than that in the high-risk group. Meanwhile, the time-dependent ROC analysis was performed and ROC curve was plotted as shown in Fig. 5B. The area under the ROC curve of the predictive model was 0.859, which indicates a moderate predictive power of the prognostic model. In the training cohort, risk score distribution curve, scatterplot of survival status and expression heatmap of the seven hub RBP genes in the high- and low- risk groups were also plotted for verifying the evaluation ability of the model (Fig. 5C-E). Furthermore, in order to test the reliability of the above conclusions, 142 patients in the validation cohort were subjected to the same analyses. The survival curve (Fig. 6A) and ROC curve (Fig. 6B) were plotted, and the area under the ROC curve was 0.698. The risk score distribution curve, scatterplot of survival status and expression heatmap of the seven hub RBP genes in the high- and low- risk groups in the validation cohort showed similar results (Fig. 6C-E). The low-risk group also presented higher survival rate compared with the high-risk group. Collectively, these demonstrated the sensitivity and specificity of the prognostic risk score model.

### Independent prognostic factors and nomogram of the seven hub RBPs

**Fig. 7:**
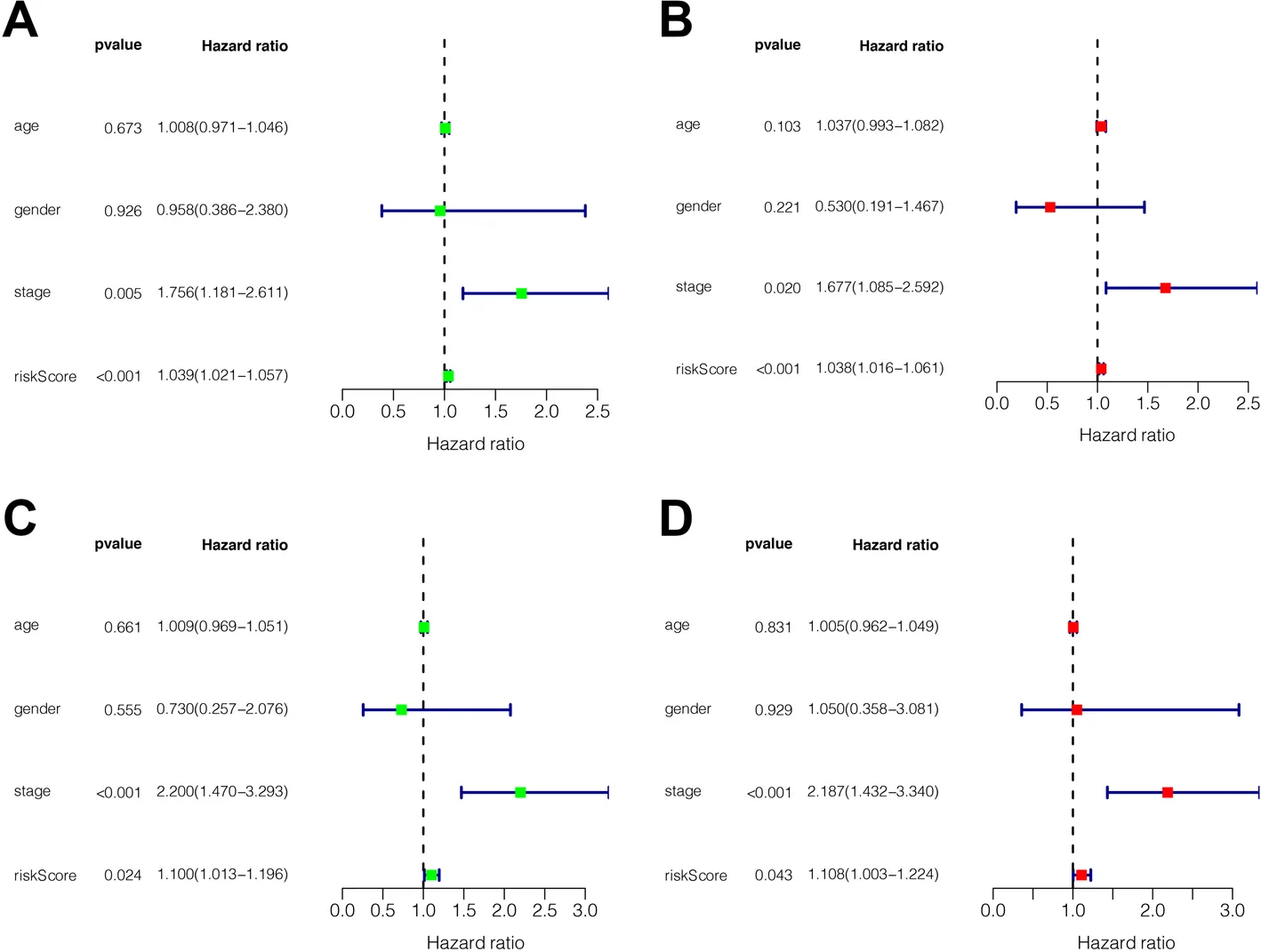
Identification of independent prognostic factors for pRCC. (A) Univariate Cox regression analysis showing the association between clinical characteristics and OS in the training cohort. (B) Multivariate Cox regression analysis identifying independent prognostic factors in the training cohort. (C) Univariate Cox regression analysis in the validation cohort. (D) Multivariate Cox regression analysis in the validation cohort.Hazard ratios (HR) and 95% confidence intervals (CI) are shown for each variable. *P < 0.05 was considered statistically significant. Both risk score and tumor grade were identified as independent prognostic factors in both cohorts

In pursuit of finding independent prognostic factors associated with the overall survival of pRCC, we conducted univariate and multivariate Cox regression analyses for some relevant clinical characteristics in the training and validation cohorts, and made the corresponding forest plots for visualization (Fig. 7). We found the significant difference between disease grade/risk score and the overall survival of pRCC patients (Both *P* < 0.05). We then performed a multivariate Cox regression analysis and found that both risk score and disease grade were independent prognostic factors related to overall survival (Both *P* < 0.05).

**Fig. 8:**
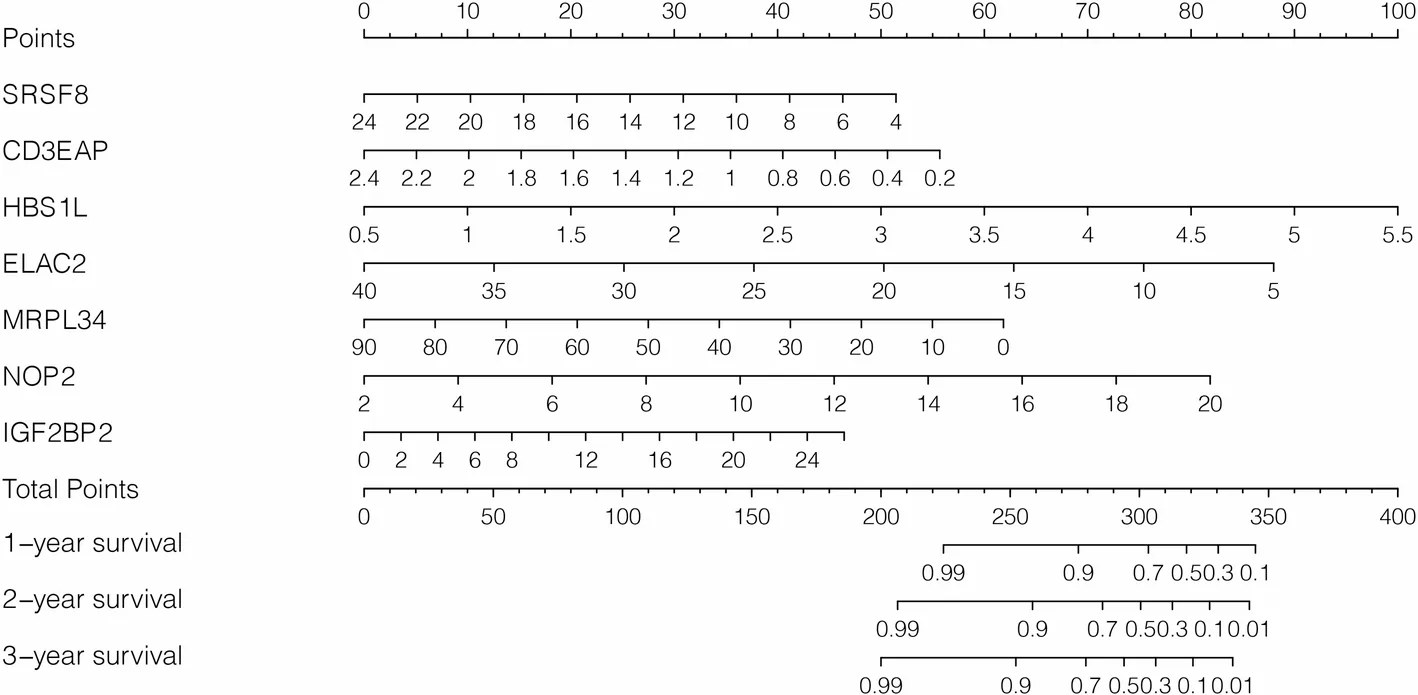
Prognostic nomogram integrating the 7-RBP risk score and clinical stage.The nomogram was constructed to predict the probability of 1-year, 2-year, and 3-year OS in pRCC patients. To use the nomogram, locate the value of each variable on the corresponding axis, draw a vertical line upward to the points axis to determine the points for each variable, sum the points for all variables, and draw a vertical line downward from the total points axis to the survival probability axes to obtain the predicted survival probabilities

In an effort to predict the survival time of pRCC patients, we in the end constructed a nomogram containing the seven prognosis-related hub RBP genes (Fig. 8). In the nomogram, each RBP gene was scored with the range of 0–100 according to its differential expression. The sum of the seven scores of each RBP could serve as the total score for predicting the survival rate of pRCC patients in the 1/2/3 years, which would help make better clinical decisions.

### Validation of predictive accuracy via Human Protein Atlas Database

**Fig. 9:**
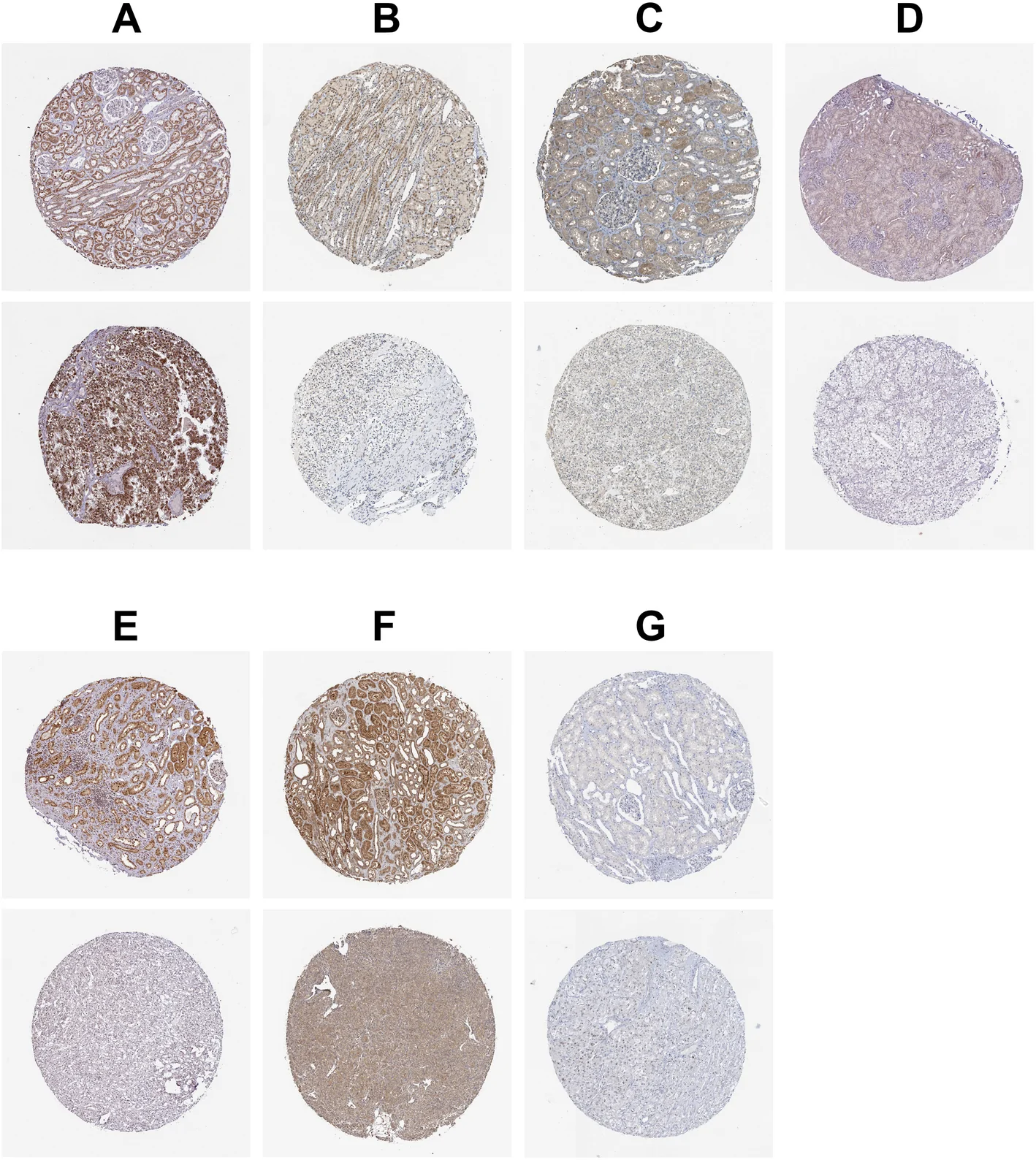
Immunohistochemical validation of the seven hub RBPs using the Human Protein Atlas database.Representative immunohistochemical staining images showing the protein expression levels of the seven hub RBPs in normal renal tissues (left panels) and pRCC tissues (right panels): (A) CD3EAP, (B) ELAC2, (C) IGF2BP2, (D) MRPL34, (E) SRSF8, (F) HBS1L, and (G) NOP2.Consistent with our transcriptomic analysis, CD3EAP was significantly upregulated in pRCC tissues, while ELAC2, IGF2BP2, MRPL34, SRSF8, and HBS1L were significantly downregulated. No significant difference was observed for NOP2

To further validate the predictive accuracy of the seven hub RBPs, immunohistochemical data of the corresponding genes in normal and tumor tissues were obtained from the Human Protein Atlas database (Fig. 9). We found CD3EAP was significantly elevated in pRCC, while the expression of ELAC2, IGF2BP2, MRPL34, SRSF8 and HBS1L was significantly reduced. Besides, no significant difference was seen in NOP2 expression between pRCC tissue and normal renal tissue.

### External validation in GEO dataset and experimental validation in cell lines

**Fig. 10:**
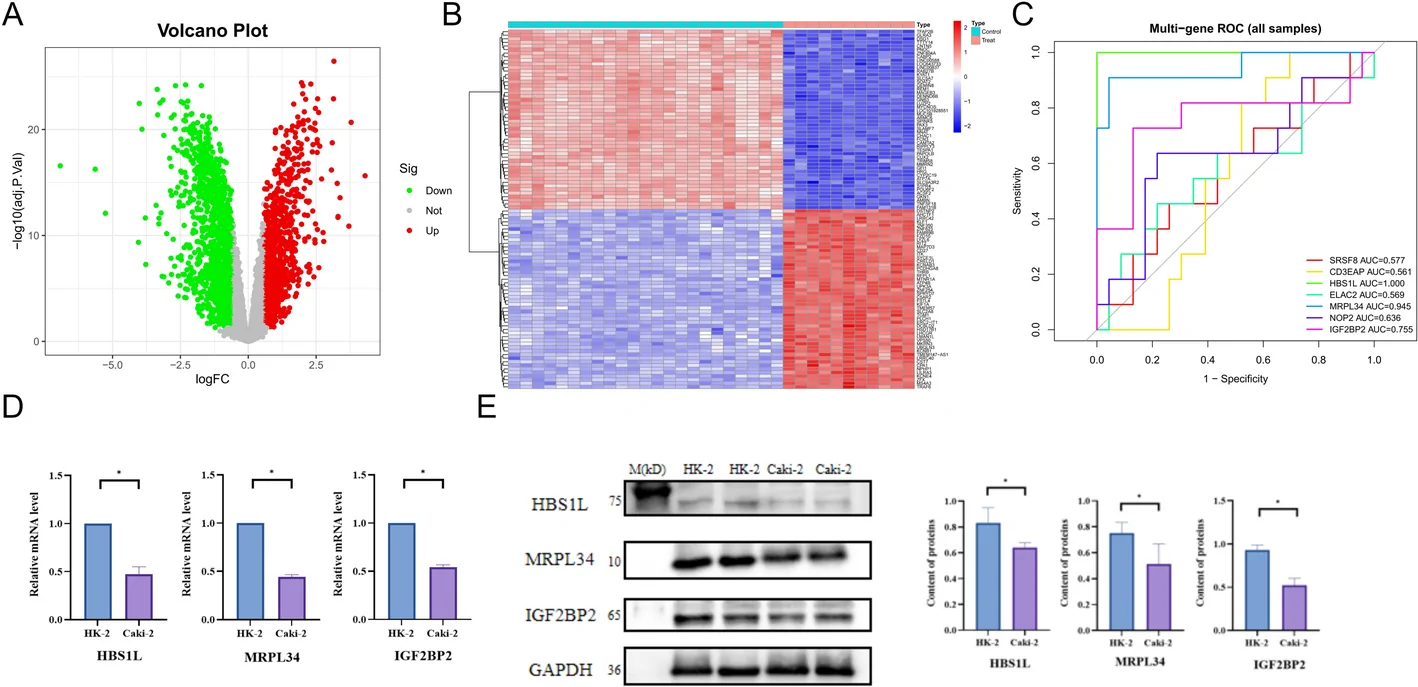
External validation in GEO dataset and experimental validation in cell lines. (**A**) Volcano plot of differentially expressed RBPs in the GSE15641 dataset; (**B**) Expression heatmap of the seven hub prognostic RBPs in the GSE15641 dataset; (**C**) Single-gene ROC curves of the seven hub RBPs; (**D**) RT-qPCR detection of mRNA expression levels of HBS1L, MRPL34 and IGF2BP2 in HK-2 and Caki-2 cells; (**E**) WB detection and quantitative analysis of protein expression levels of HBS1L, MRPL34 and IGF2BP2 in HK-2 and Caki-2 cells. *P < 0.05

To further verify the expression patterns and diagnostic value of the seven hub prognostic RBPs, we introduced the GSE15641 dataset from the GEO database for independent external validation. The volcano plot of differentially expressed RBPs showed that the expression trends of the seven hub RBPs in pRCC tissues versus normal renal tissues were consistent with those in the TCGA dataset (Fig. 10A). The expression heatmap intuitively presented the expression differences of the seven hub RBPs in the GSE15641 dataset (Fig. 10B). Single-gene ROC curve analysis showed that all seven hub RBPs had certain diagnostic efficacy for pRCC, among which HBS1L had the highest AUC value (1.000), followed by MRPL34 (0.945) and IGF2BP2 (0.755) (Fig. 10C).

Based on the above results, we selected HBS1L, MRPL34 and IGF2BP2, which showed the most significant expression differences and high diagnostic efficacy, for experimental validation at the cellular level. RT-qPCR results showed that the mRNA expression levels of HBS1L, MRPL34 and IGF2BP2 were significantly down-regulated in the pRCC cell line Caki-2 compared with the normal renal tubular epithelial cell line HK-2 (all *P* < 0.05, Fig. 10D). WB results further confirmed that the protein expression levels of HBS1L, MRPL34 and IGF2BP2 were also significantly lower in Caki-2 cells than in HK-2 cells (all *P* < 0.05, Fig. 10E), which was consistent with the bioinformatics analysis results.

## Discussion

With the advancement of high-throughput sequencing technology at unprecedented pace, a large amount of genomic data has been utilized for biomarker prediction, prognostic analysis and targeted therapy [2]. Beyond all doubt, alterations in the expression and function of RBPs could amplify the role of cancer driver genes, and promote tumor progression and aggressiveness [42]. Currently, prognosis-related RBPs have been screened in a variety of cancers such as liver cancer [58], lung cancer [33, 65], colorectal cancer [24], ovarian cancer [23] and clear cell carcinoma [55]. The investigation of prognosis-related RBPs would help better understand the molecular mechanisms of tumorigenesis and predict the prognosis of individual patients. Based on this, the unclear roles of RBPs in pRCC have constantly encouraged us to further study their biological functions in pRCC and discover the clinical significance. In this work, DERBPs were first identified from the RNA sequencing data and clinical information of pRCC and normal renal tissue samples downloaded from the TCGA database, and then received GO term and KEGG pathway enrichment anlyses. Key gene cluster modules of the selected RBPs were identified after analysis of the well-established PPI network. With the final extracted hub DERBP genes, we constructed a prognostic risk score model to assess the prognosis of pRCC patients and performed ROC analysis to evaluate the feasibility of the model. Besides, a nomogram based on the selected RBP genes was plotted for predicting the survival rate of pRCC patients. These may contribute to the design of new potential biomarkers for the treatment of pRCC and provide some reference for its diagnosis and prognosis.

GO term enrichment analysis revealed that the DERBPs were significantly enriched in ncRNA processing and metabolism, RNA splicing, catabolism, and phosphodiester bond hydrolysis, regulation of cellular amide metabolism and translation, RNA and tRNA catalytic activity, ribonuclease and nuclease activity, ribosome biogenesis, and spliceosome assembly. Accumulating evidence indicates that dysregulated RNA modification contributes to tumorigenesis and cancer progression [14]. As core components of ribonucleoprotein complexes, RBPs regulate pre-mRNA processing, mRNA transport, localization, translation, and stability [16, 39]. Increasing studies have demonstrated that RBP-RNA interactions play critical roles in cancer development and progression. For example, SORBS2 suppresses hepatocellular carcinoma tumorigenesis and metastasis by directly binding to the 3’UTR of RORA mRNA and inhibiting its degradation [22]. IGF2BP3 directly binds to the mRNAs of cyclins D1, D3, and G1, thereby upregulating their expression and promoting cancer cell proliferation [45]. QKI-5 post-transcriptionally stabilizes RASA1 mRNA by binding to its QKI response element, which in turn deactivates the Ras-MAPK signaling pathway and inhibits cell proliferation [70]. Furthermore, KEGG pathway analysis indicated that the DERBPs may regulate pRCC progression through involvement in ribosome biogenesis, spliceosome assembly, RNA polymerase activity, RNA degradation, mRNA surveillance, nucleocytoplasmic transport, and protein export.

We then constructed a PPI network based on the DERBPs and obtained 70 key RBPs from three key gene cluster modules. Although the correlation of most RBPs with pRCC is unclear, some RBPs have been reported to be closely associated with some other cancers. The mRNA helicase EIF4A1 serves a critical role in protein translation initiation, and its high intratumoral expression could facilitate epithelial-to-mesenchymal transition of gastric cancer cells and predict an unfavorable prognosis [19]. It is also the direct target for the tumor suppressor miR-1284 and could be blocked by the latter to inhibit the invasion, migration and proliferation of gastric cancer cells [60]. Besides, PHGDH could promote pancreatic cancer progression via interaction with EIF4A1 and EIF4E [35]. Overexpression of HNRNPAB would induce epithelial-mesenchymal transformation and promote metastasis of hepatocellular carcinoma through transcriptional down-regulation of SNAIL [72] and lncRNA-ELF209 [66]. And its interaction with lncRNA-PCAT19 could activate some cell-cycle genes associated with the progression of prostate cancer and thereby promote the tumor growth and metastasis [28]. TFB2M could promote cell growth and metastasis via facilitating the production of reactive oxygen species and activating Akt/NF-κB signaling in hepatocellular carcinoma [20]. GNL3, as a nucleolar GTP-binding protein that is highly expressed in various types of cancer cells, could promote the progression of colon cancer [54] and non-Hodgkin’s lymphoma [12] by activating the Wnt/β-catenin signaling. It has been reported to play an oncogenic role in the progression of osteosarcoma through regulation of epithelial-mesenchymal transition [32]. RALY is a member of the heterogeneous nuclear ribonucleoproteins, and exerts a crucial role in mRNA splicing and metabolism. Mounting studies have shown that RALY is a tumor driver in breast cancer [4], liver cancer [73], cervical cancer [9], non-small cell carcinoma [50] and pancreatic cancer [46].

Using univariate and multivariate Cox regression analyses combined with survival analysis, we identified seven hub RBPs associated with pRCC prognosis: SRSF8, CD3EAP, HBS1L, ELAC2, MRPL34, NOP2, and IGF2BP2. Most of the RBPs are reported to be related to the occurrence, progression and prognosis of malignancies. SRSF8 may be used as a prognostic biomarker for breast cancer [69] and prostate cancer [7]. CD3EAP is associated with the prognosis of soft tissue sarcoma [34] and could predict the survival rate of patients with endometrial cancer [62]. ELAC2 has been considered an important genetic factor that can increase the risk of prostate cancer in several populations [1], and its nuclear overexpression would escalate the risk of relapse after radical prostatectomy [47]. IGF2BP2 holds an important role RNA localization, stability and translation. Its upregulation activates the PI3K/Akt signaling to promote the cell proliferation in pancreatic carcinoma [63], and its knockdown could significantly reduce the cell proliferation of ovarian high-grade serous carcinomas [26]. IGF2BP2 may be used as a prognostic biomarker and potential new therapeutic target for acute myeloid leukemia [25]. Its presence has been widely reported in various malignancies such as oral cancer [8], colorectal cancer [11, 27, 59], liver cancer [18, 43], breast cancer [37], lung cancer [29] and thyroid cancer [15]. NOP2 is associated with the metastasis in prostate cancer [52, 53], and its up-regulation by lncRNA-hPVT1 via stabilization of its protein could promote cell proliferation in hepatocellular carcinoma [56]. Besides, NOP2 could serve as a potential prognostic predictor for clear cell RCC [57, 58].

A remarkable and novel finding of this study is that the seven core prognostic RNA-binding proteins (RBPs) predominantly exhibit a downregulated expression pattern in papillary renal cell carcinoma (pRCC) tissues, with only CD3EAP showing significant upregulation. This expression profile differs markedly from the characteristic pattern observed in most other cancers, where oncogenic RBPs are generally overexpressed, suggesting a unique role of post-transcriptional regulation in the pathogenesis of pRCC. Specifically, CD3EAP, the only upregulated core RBP, has been previously confirmed to be associated with poor prognosis in soft tissue sarcoma and endometrial cancer, and its overexpression in pRCC may promote tumor progression by enhancing ribosome biogenesis and protein synthesis. Conversely, the consistently downregulated expression of HBS1L, MRPL34, and IGF2BP2, all validated by our RT-qPCR and Western blot experiments, indicates that these RBPs may function as tumor suppressors in pRCC. Furthermore, the downregulation of SRSF8 (involved in RNA splicing) and ELAC2 (involved in tRNA processing) in pRCC may lead to aberrant splicing of tumor suppressor genes or impaired tRNA maturation, thereby contributing to tumorigenesis. Whereas NOP2, a nucleolar protein involved in rRNA modification, was identified as an independent prognostic factor despite showing no significant expression difference between tumor and normal tissues, suggesting that it may exert its prognostic effect through post-translational modifications or protein-protein interactions rather than changes in mRNA expression—a mechanism that warrants further in-depth investigation in pRCC.

Collectively, while these prognostic RBPs have been implicated in various malignancies, our findings suggest that they also play critical roles in pRCC and may provide valuable insights for the diagnosis and management of this disease. We subsequently constructed a prognostic risk score model based on TCGA data, and validation analyses demonstrated that these seven RBP genes have good prognostic performance. The prognostic value of our model was further confirmed using data from the Human Protein Atlas database. Notably, the specific molecular mechanisms by which these seven hub RBPs contribute to pRCC pathogenesis remain largely unexplored. Therefore, further mechanistic studies are needed to elucidate their roles in pRCC and identify potential therapeutic targets. Finally, we constructed a nomogram incorporating the seven hub RBPs to assist clinicians in predicting patient survival and developing individualized treatment strategies for pRCC.

The 7-RNA-binding protein (RBP) prognostic model constructed in this study not only exhibited excellent prognostic prediction performance in the internal TCGA training and validation cohorts, but also further confirmed its clinical application value and innovation through systematic comparison with existing clinicopathological prognostic systems and published molecular prognostic models [77]; in terms of clinicopathological models, the currently widely used prognostic assessment systems for papillary renal cell carcinoma (PRCC) include TNM staging, UISS score, Leibovich model, and the VENUSS scoring system specifically developed for PRCC [75], all of which are constructed based on clinicopathological features such as tumor size, stage, nuclear grade, and venous tumor thrombus, and while they are easy to promote in clinical practice, they have obvious limitations: first, the assessment of pathological features is susceptible to the subjective judgment of pathologists, and there is particularly large inter-observer variability in the determination of nuclear grade and sarcomatoid differentiation; second, these models cannot explain the molecular heterogeneity of PRCC, making it difficult to achieve accurate stratification for patients with the same clinical stage but significantly different prognoses. The RBP molecular model constructed in this study is based on objective gene expression data and can identify high-risk patients at the molecular level, with AUC values for predicting 3-year and 5-year overall survival significantly higher than those of TNM staging and Leibovich model and comparable to the predictive performance of the VENUSS scoring system, while simultaneously providing clues to the biological mechanisms underlying abnormal post-transcriptional regulation and pointing out the direction for the development of subsequent targeted therapies. Compared with the PRCC-related RBP prognostic study published by Jiang et al., in 2021 [76], this study has significant differences and has achieved important progress in multiple aspects: first, there is no overlap at all in the prognostic-related RBP genes screened by the two studies, as the model of Jiang et al., includes 6 genes such as SNRPN, RRS1, and INTS8, while this study identified 7 novel prognostic-related RBPs including CD3EAP, ELAC2, IGF2BP2, MRPL34, SRSF8, HBS1L, and NOP2, further enriching the prognostic molecular marker library of PRCC; second, the validation system of this study is more comprehensive and rigorous, as this study not only completed cross-validation within TCGA, but also performed external validation using the GSE15641 independent cohort from the GEO database, and meanwhile verified the differential expressions of HBS1L, MRPL34, and IGF2BP2 in human normal renal tubular epithelial cell line HK-2 and PRCC cell line Caki-2 by RT-qPCR and Western blot experiments, providing a solid experimental basis for the reliability of the model; third, this study has higher clinical translation value, as we constructed a nomogram integrating RBP risk score and clinical stage, and confirmed through calibration curves that its prediction results are highly consistent with the actual survival outcomes, which can provide clinicians with individualized survival probability prediction. Furthermore, compared with the published PRCC immune-related prognostic models [79, 80] and cuproptosis-related lncRNA prognostic model [78], this study systematically analyzed the expression profile and prognostic value of RBPs in PRCC, revealed the important role of abnormal RNA post-transcriptional regulation in the occurrence and development of PRCC, and provided a new perspective for the molecular mechanism research of PRCC. Collectively, the 7-RBP prognostic model constructed in this study is superior to existing similar studies in terms of gene composition, validation system, clinical practicality, and generalization ability, providing new tools and targets for the prognostic assessment and individualized treatment of PRCC.

Our study has verified the expression patterns of core RBPs through the GSE15641 independent dataset and cell line experiments, and performed rigorous internal validation of the prognostic model using a hold-out method. However, there are still the following limitations: First, our study lacks independent external validation of the prognostic model using a separate pRCC cohort with complete survival information. The GSE15641 dataset was only used for expression and diagnostic validation due to the lack of survival data. Future studies with larger multi-center clinical cohorts are needed to further confirm the generalizability of our prognostic model. Second, while our stepwise feature selection strategy and rigorous internal validation have minimized the risk of overfitting, more advanced feature selection methods such as LASSO-Cox regression could be employed in future studies to further optimize the model and improve its generalizability. Third, the relatively limited clinical information of TCGA data may affect the comprehensive predictive ability of the model. Fourth, the specific molecular mechanisms of core RBPs in pRCC still need to be further clarified through in vitro and in vivo functional experiments, including cell proliferation, migration, invasion assays and animal model studies.

## Conclusion

We identified seven hub DERBPs (SRSF8, CD3EAP, HBS1L, ELAC2, MRPL34, NOP2 and IGF2BP2) in pRCC and analyzed their associated biological processes and pathways through bioinformatics analysis. These RBPs may be involved in the development, progression, invasion and metastasis of pRCC. A prognostic model constructed based on the seven hub DERBPs had good predictive power for pRCC prognosis. Our study focused on the prognostic value of RBPs in pRCC for the first time, based on previous reports of RBPs in malignancies, which is of great value for further research on the molecular mechanism in pRCC and development of potential new targeted drugs.

## Supplementary Information


Supplementary Material 1.



Supplementary Material 2.



Supplementary Material 3.



Supplementary Material 4.



Supplementary Material 5.



Supplementary Material 6.



Supplementary Material 7.



Supplementary Material 8.



Supplementary Material 9.


## Data Availability

The datasets analyzed in this study were obtained from the following public secondary databases:1.The Cancer Genome Atlas (TCGA): Papillary renal cell carcinoma (pRCC) RNA-seq and clinical data were downloaded from the TCGA-GDC portal (https://portal.gdc.cancer.gov/), project ID: TCGA-KIRP.2.STRING database: Protein–protein interaction (PPI) analysis was performed using STRING v11.5 (https://string-db.org/)0.3.The Human Protein Atlas (HPA): Immunohistochemical validation data were obtained from the Human Protein Atlas database (https://www.proteinatlas.org/).All data used in this study are publicly available and de-identified. Accession numbers and dataset identifiers are provided as above for reproducibility.
